# Increased insulin sensitivity and diminished pancreatic beta-cell function in DNA repair deficient *Ercc1*^*d*/−^ mice

**DOI:** 10.1016/j.metabol.2021.154711

**Published:** 2021-01-23

**Authors:** Ana P. Huerta Guevara, Sara J. McGowan, Melissa Kazantzis, Tania Rozgaja Stallons, Tokio Sano, Niels L. Mulder, Angelika Jurdzinski, Theo H. van Dijk, Bart J.L. Eggen, Johan W. Jonker, Laura J. Niedernhofer, Janine K. Kruit

**Affiliations:** a Sectionof Molecular Metabolism and Nutrition, Department of Pediatrics, University of Groningen, University Medical Center Groningen, Hanzeplein 1, 9700 RB Groningen, the Nethelands; b Institute on the Biology of Aging and Metabolism and Department of Biochemistry, Molecular Biology and Biophysics, University of Minnesota, 6-155 Jackson Hall, 321 Church St., Minneapolis, MN 55455, USA; c Department of Metabolism and Aging, Scripps Research Institute, Jupiter, FL 33458, USA; d Laboratory Medicine, University of Groningen, University Medical Center Groningen, Hanzeplein 1, 9700 RB Groningen, the Netherlands; e Department of Biomedical Sciences of Cells & Systems, University of Groningen, University Medical Center Groningen, Hanzeplein 1, 9700 RB Groningen, the Netherlands; f Metabolic Core, Scripps Research Institute, Jupiter, FL 33458, USA

**Keywords:** DNA repair, Energy metabolism, Glucose homeostasis, Beta-cell function, Genotoxic stress, Somatotropic axis

## Abstract

**Background:**

Type 2 diabetes (T2DM) is an age-associated disease characterized by hyperglycemia due to insulin resistance and decreased beta-cell function. DNA damage accumulation has been associated with T2DM, but whether DNA damage plays a role in the pathogenesis of the disease is unclear. Here, we used mice deficient for the DNA excision-repair gene *Ercc1* to study the impact of persistent endogenous DNA damage accumulation on energy metabolism, glucose homeostasis and beta-cell function.

**Methods:**

ERCC1-XPF is an endonuclease required for multiple DNA repair pathways and reduced expression of ERCC1-XPF causes accelerated accumulation of unrepaired endogenous DNA damage and accelerated aging in humans and mice. In this study, energy metabolism, glucose metabolism, beta-cell function and insulin sensitivity were studied in *Ercc1*^*d*/−^ mice, which model a human progeroid syndrome.

**Results:**

*Ercc1*^*d*/−^ mice displayed suppression of the somatotropic axis and altered energy metabolism. Insulin sensitivity was increased, whereas, plasma insulin levels were decreased in *Ercc1*^*d*/−^ mice. Fasting induced hypoglycemia in *Ercc1*^*d*/−^ mice, which was the result of increased glucose disposal. *Ercc1*^*d*/−^ mice exhibit a significantly reduced beta-cell area, even compared to control mice of similar weight. Glucose-stimulated insulin secretion *in vivo* was decreased in *Ercc1*^*d*/−^ mice. Islets isolated from *Ercc1*^*d*/−^ mice showed increased DNA damage markers, decreased glucose-stimulated insulin secretion and increased susceptibility to apoptosis.

**Conclusion:**

Spontaneous DNA damage accumulation triggers an adaptive response resulting in improved insulin sensitivity. Loss of DNA repair, however, does negatively impacts beta-cell survival and function in *Ercc1*^*d*/−^ mice.

## 1. Introduction

Nuclear DNA is continuously exposed to a variety of genotoxic insults from endogenous and exogenous origins. To ensure genomic integrity and maintenance of cellular function, cells have evolved highly sophisticated DNA repair mechanisms that recognize and remove specific types of DNA damage. The repair process is embedded in the DNA damage response (DDR) that activates appropriate cellular responses, including cell cycle arrest in order to allow time for repair of DNA damage, apoptosis or permanent withdrawal of cell cycle progression to minimize the detrimental effects of unrepaired DNA lesions. Although the DDR protects the organism against tumorogenesis, it is now evident that the accumulation of senescent cells, the reduction of regenerative capacity, and the induction of metabolic changes promoted by the DDR contribute to aging and to the development of age-related diseases [1].

Products of cellular metabolism are a major source of endogenous DNA damage. During mitochondrial-based aerobic metabolism, reactive oxygen species (ROS) are generated, which can modify proteins, react with lipid bilayers and damage nucleic acids. In light of this, it is not surprising that the DDR orchestrates cellular metabolism in order to avoid further genomic instability. The main regulator of DDR, the transcription factor p53, plays a key role in regulating metabolic homeostasis by decreasing glycolytic flux and promoting mitochondrial respiration [2]. In addition, the DDR stimulates the pentose phosphate pathway to promote production of the anti-oxidant cofactor NADPH and to increase nucleotide production needed for DNA repair [3]. At the organismal level, modulations of DDR or DNA repair genes in mice result in a whole range of metabolic abnormalities. Loss of DNA repair of oxidized base lesions due to deficiencies in DNA glycosylase NEIL1, 8-oxoguanine DNA glycosylase OGG1 or DNA polymerase η result in obesity, hyperinsulinemia and hyperglycemia [4–6]. Excessive and sustained p53 activation, due to overexpression of mutant p53 or loss of the ubiquitin ligase ARF-bp, results in diabetes [7–9], whereas inhibition of p53 activity improves insulin sensitivity in diabetic mice [10]. Loss of double-strand break repair and p53 results in a severe diabetic phenotype due to the depletion of insulin-producing beta-cells [11]. Collectively, these data indicate that sustained signaling through the DDR impacts glucose metabolism. As aging increases the burden of DNA lesions [12] even in the face of normal DNA repair [13], sustained DDR signaling could play a causal role in the pathogenesis of age-related metabolic diseases such as type 2 diabetes (T2DM).

Interestingly, despite clear evidence of DNA damage accumulation in multiple tissues [13], mice deficient in transcription-coupled nucleotide excision repair (TC-NER) do not develop T2DM and even show hypoglycemia [14,15]. Loss of the TC-NER pathway is causally linked to Cockayne syndrome, a rare and often severe progeroid disorder characterized by growth failure, progressive neurological abnormalities, age-related organ dysfunction and shortened life expectancy [16]. Defects in TC-NER lead to suppression of the growth hormone (GH)/insulin like growth factor (IGF1) axis, suggesting that persistent DNA damage contributes to the aging-associated shift from growth to somatic maintenance [14,17,18], which is accelerated when DNA repair is attenuated. Suppression of the somatotropic axis in TC-NER deficient mice could potentially have beneficial metabolic effects as suppression of the GH/IGF axis is associated with enhanced insulin sensitivity [19,20] and lifespan extension [21]. To determine whether this survival response could counteract the detrimental effects of DNA damage accumulation on glucose metabolism, we studied insulin sensitivity, glucose homeostasis and beta-cell function in mice deficient in the DNA excision repair gene *Ercc1*.

## 2. Material and methods

### 2.1. Animals

*Ercc1*^*d*/−^ and littermate controls were generated in a F1 hybrid background by crossing C57BL/6J and FVB/N mice as previously described [22]. *Ercc1*^+/−^ and *Ercc1*^*d*/+^ mice displayed a wild-type phenotype and were used as littermate controls. For this study, male mice were used at age 4–16 weeks. Animals were group housed in a light- and temperature-controlled facility (lights on from 7 a.m. to 7 p.m., 21 °C) with free access to water and standard chow (SDS diets RM3). All experiments were approved by the Institutional Animal Care and Use Committees at the University of Groningen, the Netherlands or Scripps Research, FL, USA.

### 2.2. Animal experiments and hormone analysis

Glucose levels were determined using OneTouch Ultra blood glucose meter (LifeScan Benelux, Belgium). Plasma insulin, glucagon and IGF1 levels were measured using an ELISA kit (Crystal Chem, Diagnostic Systems Laboratories Inc., Texas, United States). Epinephrine was measured using in matrix derivatization combined with isotope dilution liquid chromatography tandem mass spectrometry (LC-MS/MS) as previously described [23]. HOMA-IR was calculated using 10-hour fasted blood glucose and insulin levels as previously described [24]. Glucose tolerance tests were performed on 10-hour fasted mice after the administration of 2 g glucose/kg body weight orally. Insulin tolerance tests were performed on 4-hour fasted mice i.p. injected with 0,25 unit of insulin (Novorapid, Novo Nordisk, Denmark) per kg body weight. Kinetic parameters including hepatic insulin sensitivity and peripheral insulin sensitivity were calculated after the administration of the [6,6-^2^H_2_]glucose tracer as previously described [24]. Glucagon tests were performed on non-fasted mice injected with 1 mg of glucagon (Sigma Aldrich) per kg body weight. For tissue collection, 12-week-old mice were anesthetized with isoflurane and euthanized by cardiac puncture. Tissues were collected, snap-frozen in liquid nitrogen and stored at −80 °C or processed for histology.

### 2.3. Indirect calorimetry

Real-time metabolic analyses were performed using a Comprehensive Laboratory Animal Monitoring System (CLAMS, Columbus Instruments). After a period of 3 days of acclimatization, CO_2_ production, O_2_ consumption, respiratory exchange ratio (RER) and activity were determined in the presence of food. Body composition was measured using nuclear magnetic resonance (LP50 BCA-analyzer; Bruker Optics). Energy expenditure was calculated based on O_2_ consumption and CO_2_ production and analyzed by ANCOVA using lean mass as covariate [25].

### 2.4. Primary mouse islet isolation, cell culture and in vitro assays

Islets were isolated by collagenase digestion as previously described [26]. Islets were rinsed and handpicked in RPMI media containing 10% FBS after which islets were frozen immediately for RNA isolation or cultured overnight. To determine glucose-induced insulin secretion, cultured islets were size matched after which the insulin secretion assay was performed as previously described [27]. Insulin levels in media and islets were measured by ELISA (Mouse-Insulin Ultra Sensitive ELISA Alpco, Salem, NH, USA). Islet protein levels were measured by the Bradford method. For the glucotoxicity measurement, islets were cultured for 7 days in RPMI media containing 10% FBS with 11 mM or 33 mM glucose. Cell death was determined by fluorescence microscopy after Hoechst 33342 (Sigma-Aldrich) and propidium iodide (Sigma-Aldrich) staining [28].

### 2.5. Histology and immunostaining

Formalin-fixed pancreatic tissues were embedded in paraffin, sectioned, deparaffinized and rehydrated using standard techniques. For immunofluorescence, sections were incubated overnight at 4 °C with antibodies against insulin (Abcam, Cambridge, UK), glucagon (Dako, Glostrup, Denmark), and/or Ki67 (Abcam, Cambridge, UK), followed by secondary antibodies conjugated to FITC or Cy3 (Life Technologies). DAPI-containing mounting media (Vector Laboratories, Burlingame, CA, USA) was added to coverslips. Apoptotic cells were identified by the TUNEL technique (Roche). Immunofluorescence staining was quantified using ImageJ. For quantification, all the islets embedded in 2 pancreatic sections separated by 200 μm were analyzed, resulting in the counting of at least 500 beta-cells/mouse. For beta-cell area measurements, the percentage of insulin-positive surface area was determined in 8 evenly spaced slices of pancreas using ImageScope (Aperio).

### 2.6. Gene expression analysis

Total RNA from isolated islets and liver tissue was isolated using Trizol (Life Technologies) after which cDNA was synthesized using Moloney-Murine Leukemia Virus (M-MLV) reverse transcriptase (RT) (Life Technologies) with random primers. SYBR Green PCR Master Mix (Life Technologies) or FAST PCR mix and Taqman probes (Applied Biosystems Europe) with a 7900HT FAST system. Expression values were normalized to beta-actin and 36B4 mRNA levels.

### 2.7. Statistical analysis

Graphpad Prism 8.0 was used for statistical analysis. Data are presented as Tukey’s Box-and-Whiskers plots using median and 25th and 75th percentile intervals (P_25_–P_75_) or means ± standard deviation for the glucose and insulin tolerance tests. Differences between groups were calculated by Mann-Whitney test with a *P* value of 0.05 considered significant. A repeated measurement two-way ANOVA, followed by Bonferroni posthoc tests, was used to evaluate the insulin tolerance, glucagon stimulation and glucose tolerance tests. The ANCOVA analysis of the energy expenditure was done using SPSS v25.

## 3. Results

### 3.1. Ercc1^d/−^ mice show reduced body weight, increased activity and altered energetics

**Fig. 1.**
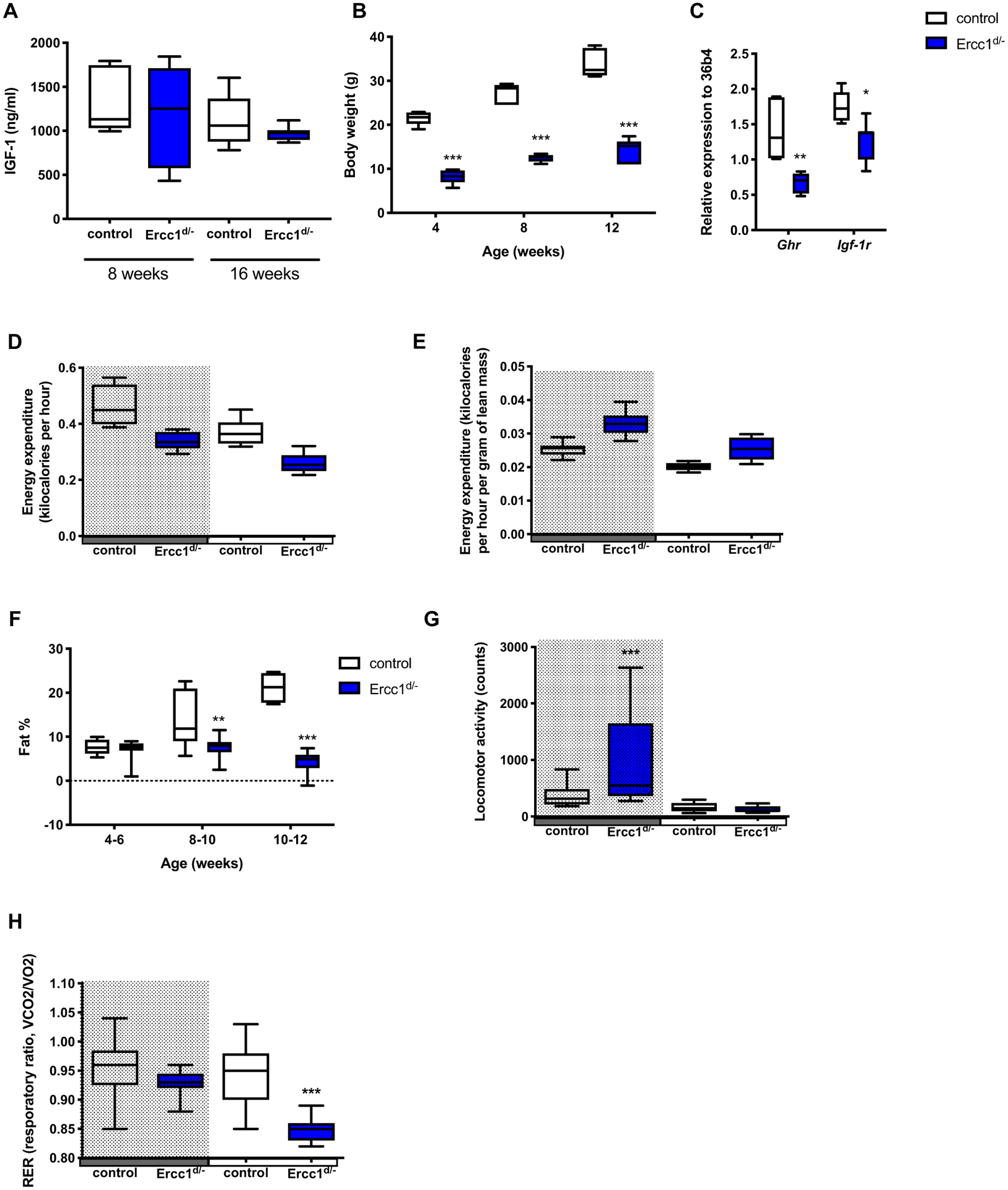
*Ercc1*^*d*/−^ mice show attenuation of the somatotropic axis, increased activity and altered substrate utilization. (A) Plasma IGF-1 levels of *Ercc1*^*d*/−^ mice and littermate controls (n = 6–9 mice per group). (B) Body weight of *Ercc1*^*d*/−^ mice and controls (n = 5–6 mice per group). (C) Expression of growth hormone receptor (*Ghr*) and Igf-1 receptor (*Igf-1r*) in liver of *Ercc1*^*d*/−^ mice and age-matched controls (n = 5–6 mice per group). Mice of 9 weeks of age (n = 9 mice per group) were housed in CLAMS cages. After 3 days of acclimatization, (D) energy expenditure per mouse or (E) adjusted energy expenditure for lean mass, (G) locomotor activity, and (H) respiratory exchange ratio (RER) were measured during the dark (grey) and light period. (F) Fat percentage of *Ercc1*^*d*/−^ mice and controls at different ages. *P* < 0.05*, *P* < 0.01**, *P* < 0.001*** by Mann-Whitney test.

Total ablation of *Ercc1* in mice (*Ercc1*^−/−^ mice) results in a dramatic reduction in plasma IGF-1 levels, growth retardation and a reduced lifespan of 4 weeks [14]. *Ercc1* hypomorphic mice (*Ercc1*^*d*/−^), expressing one *Ercc1* null allele and one mutant *Ercc1* allele carrying a 7 amino acid deletion at the C-terminus of the protein, better mimic the human XFE progeroid syndrome caused by reduced expression of ERCC1-XPF, and develop healthy until early adulthood, after which rapid, progressive, premature aging occurs [29]. *Ercc1*^*d*/−^ mice show accelerated accumulation of DNA damage and senescent cells in multiple tissues [13,30,31] and were therefore chosen as a model of NER deficiency. Although *Ercc1*^*d*/−^ mice did not show significant changes in plasma IGF-1 levels (Fig. 1A), body weight was significantly reduced (Fig. 1B). This difference in size was accompanied by decreased expression of the *growth hormone receptor* (*Ghr*) and *Igf-1 receptor* (*Igf-1r*) in the liver (Fig. 1C), demonstrating suppression of the GH/IGF-1 axis of *Ercc1*^*d*/−^ mice, similar to *Ercc1*^−/−^ mice [14], downstream or as a consequence of chronic genotoxic stress.

Energy expenditure not corrected for body weight or lean mass was decreased in mice deficient for *Ercc1* (Fig. 1D), similar to mice with suppressed GH/IGF-1 axis [32]. As fat percentage was significantly reduced at 8 weeks of age in *Ercc1*^*d*/−^ mice (Fig. 1F), correcting energy expenditure for lean mass revealed in increased energy expenditure in *Ercc1*^*d*/−^ mice (Fig. 1E). Energy expenditure values adjusted for the variation in lean mass using multiple linear regression analysis (ANCOVA) revealed no difference between the genotypes (*P* = 0.9). *Ercc1*^*d*/−^ mice did show increased locomotor activity during the dark phase (Fig. 1G). The respiratory exchange ratio (RER) was similar when comparing *Ercc1*^*d*/−^ mice and controls during the dark phase; however, upon the light phase, the RER of *Ercc1*^*d*/−^ mice decreased rapidly (Fig. 1H), indicating a shift in substrate utilization from carbohydrates to fat.

### 3.2. Reduced Ercc1 expression results in fasting hypoglycemia and increased insulin sensitivity

**Fig. 2.**
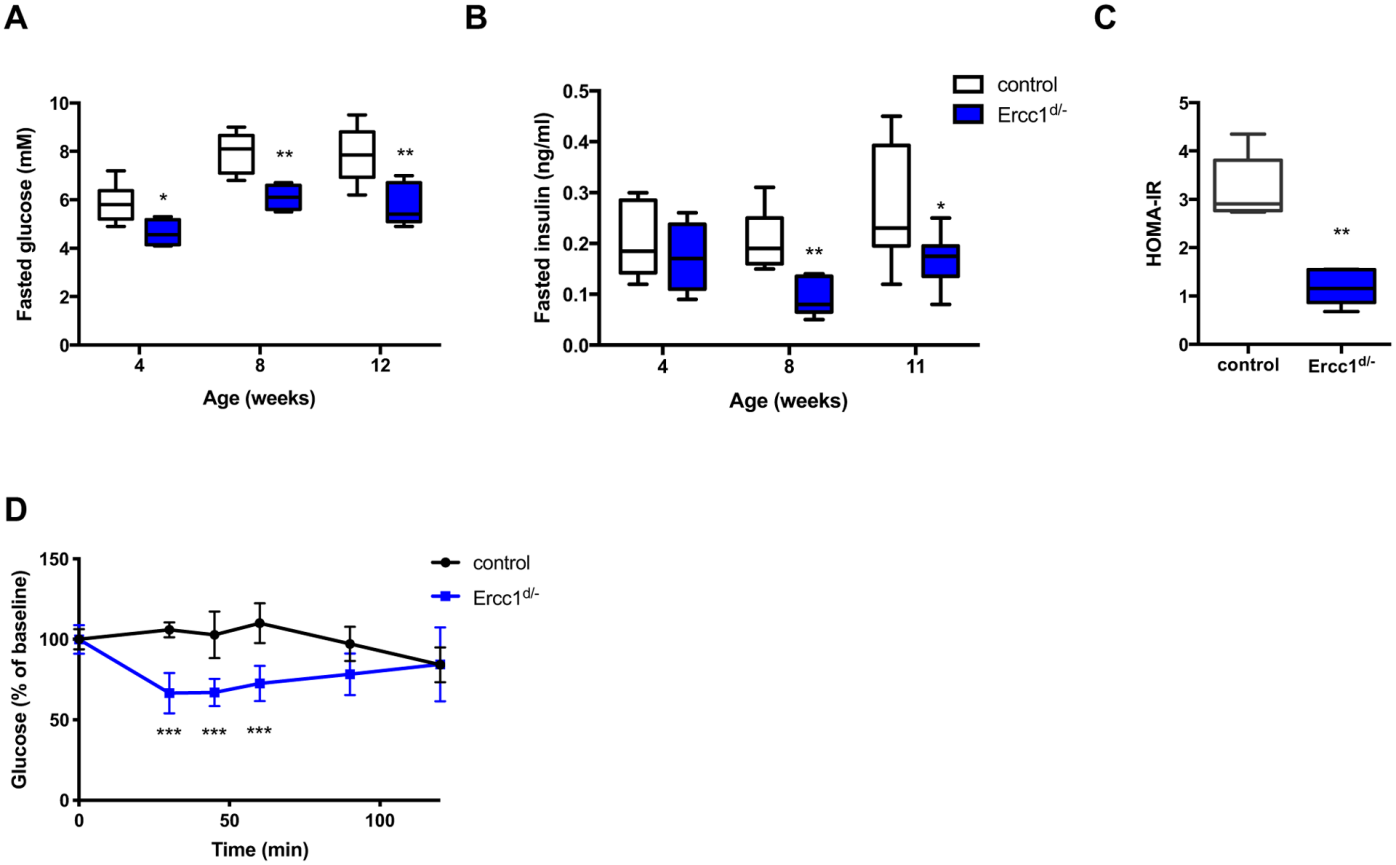
*Ercc1*^*d*/−^ mice display hypoglycemia and hypoinsulinemia (A) Fasted blood glucose levels of *Ercc1*^*d*/−^ mice and controls at multiple ages (n = 5–8 mice per group). (B) Plasma insulin levels were measured in fasted *Ercc1*^*d*/−^ mice and age-matched controls (n = 5–8 mice per group). (C) HOMA-IR (insulin resistance) was calculated using the measurements for fasted glucose and insulin levels (n = 8 mice per group). (D) Insulin tolerance test was performed on *Ercc1*^*d*/−^ mice and controls fasted for 4 h (n = 6 mice per group) using a 0.25 U/kg body weight dose. *P* < 0.05*, *P* < 0.01**, *P* < 0.001***.

**Fig. 3.**
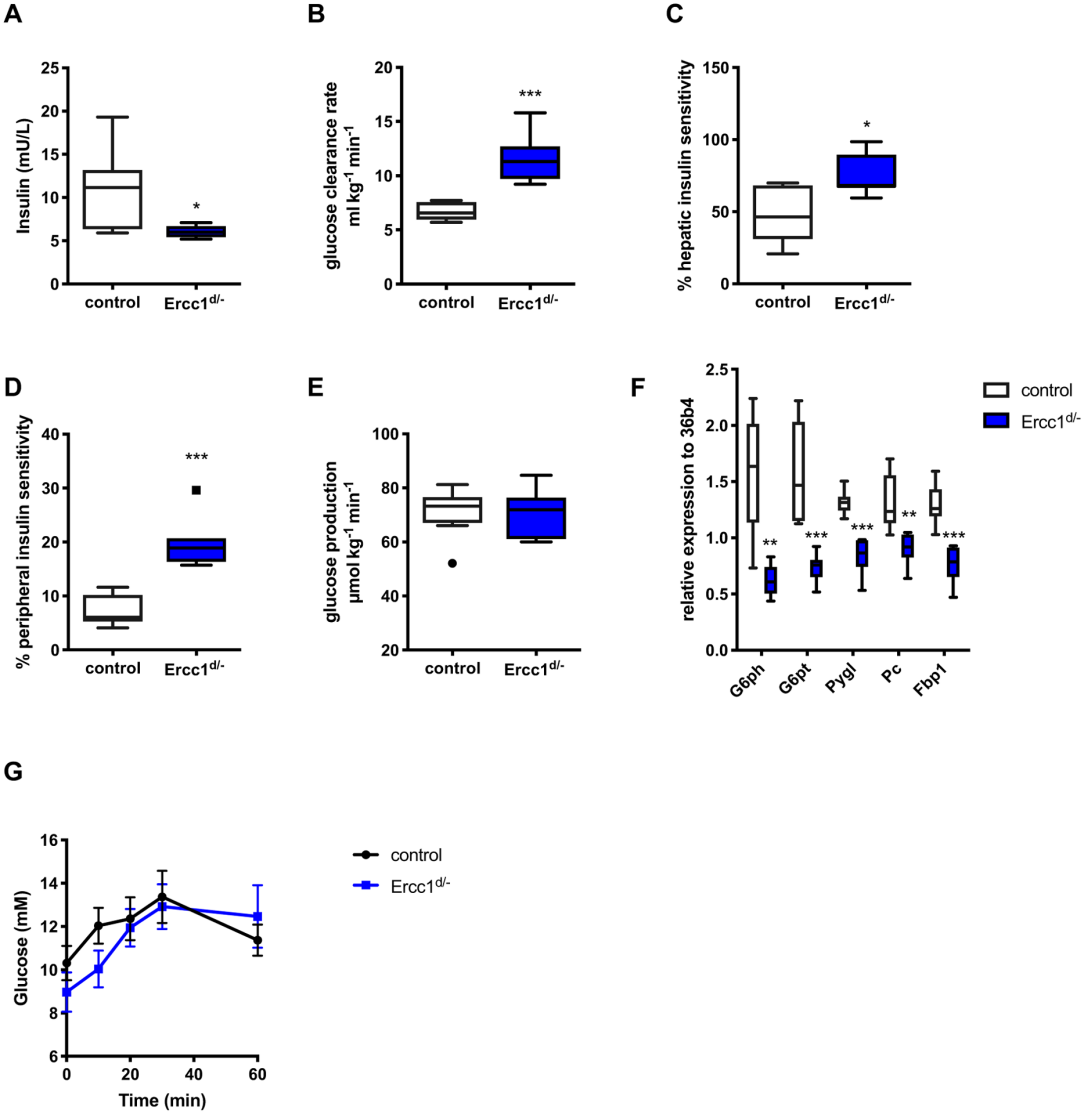
Mice with reduced *Ercc1* expression show improved glucose clearance and increased peripheral insulin sensitivity. (A) Plasma insulin levels were measured in fasted *Ercc1*^*d*/−^ mice and age-matched controls (n = 8 mice per group). *Ercc1*^*d*/−^ mice and controls (n = 8 mice per group) received an injection of [6,6-^2^H_2_] glucose tracer to calculate the following kinetic parameters: (B) glucose clearance rate, (C) hepatic insulin sensitivity, (D) peripheral insulin sensitivity and (E) glucose production. (F) Relative expression of genes involved in glycogenolysis, gluconeogenesis and glucose production in the liver of 12 weeks old *Ercc1*^*d*/−^ mice and controls (n = 7 mice per group). Glucagon was administered (G) to *Ercc1*^*d*/−^ mice and controls (n = 6 mice per group) to assess glucose production. *P* = 0.05^#^, *P* < 0.05*, *P* < 0.01**, *P* < 0.001***.

*Ercc1*^*d*/−^ mice showed decreased fasting glucose and insulin levels (Fig. 2A, B) starting from the age of 4 and 8 weeks, respectively. Decreased basal glucose and insulin levels suggest increased insulin sensitivity. This was confirmed in *Ercc1*^*d*/−^ mice by decreased HOMA-IR levels (Fig. 2C) and improved performance in insulin tolerance tests (Fig. 2D) using low amounts of insulin (0.25 U/kg body weight). In order to study blood glucose kinetics during fasted steady-state conditions, fasted mice received a trace amount of [6,6-^2^H_2_]glucose and the decay of the glucose label in the blood was followed over time to calculate glucose kinetics [24]. Despite lower fasting insulin levels (Fig. 3A), *Ercc1*^*d*/−^ mice showed an increase in glucose clearance rate as compared to littermate controls (Fig. 3B). Hepatic insulin sensitivity was improved in *Ercc1*^*d*/−^ mice by 46% compared to wild-type, age-matched controls (Fig. 3C), whereas peripheral insulin sensitivity was increased by 212% in *Ercc1*^*d*/−^ mice (Fig. 3D). Interestingly, glucose production was not different between *Ercc1*^*d*/−^ and control mice (Fig. 3E).

During fasting, blood glucose levels are initially maintained by optimizing endogenous glucose production by the breakdown of glycogen stores in the liver. Expression of genes involved in glucose production such as *glucose-6-phosphatase* (*G6pc*) and *glucose-6-phosphate transporter* (*G6pt*) were decreased in livers of *Ercc1*^*d*/−^ mice (Fig. 3F). In the glycogenolysis pathway, *glycogen phosphorylase* (*Pygl*), which catalyzes the hydrolysis of glycogen, was decreased in *Ercc1*^*d*/−^ mice (Fig. 3F). Furthermore, loss of *Ercc1* resulted in decreased expression of *pyruvate carboxylase* (*Pc*) and *fructose-1,6-biphosphatase 1* (*Fbp1*), which have important roles in gluconeogenesis (Fig. 3F). Surprisingly, despite major differences in expression of glycogenolysis genes, *Ercc1*^*d*/−^ mice showed no impairment in glucagon-stimulated increase in plasma glucose levels (Fig. 3G). Furthermore, glucagon and epinephrine were similar in fasted *Ercc1*^*d*/−^ and control mice (glucagon: 309 ± 18 pg/mL in control mice *vs*. 303 ± 29 pg/mL in *Ercc1*^*d*/−^ mice, epinephrine: 0.73 ± 0.16 nM in control mice *vs*. 0.79 ± 0.12 nM in *Ercc1*^*d*/−^ mice). These results suggest that the hypoglycemia observed in *Ercc1*^*d*/−^ mice is not caused by defective glucose production but caused by increased glucose uptake by peripheral tissues.

### 3.3. Decreased beta-cell function and increased levels of DNA damage markers in islets of Ercc1^d/−^ mice

**Fig. 4.**
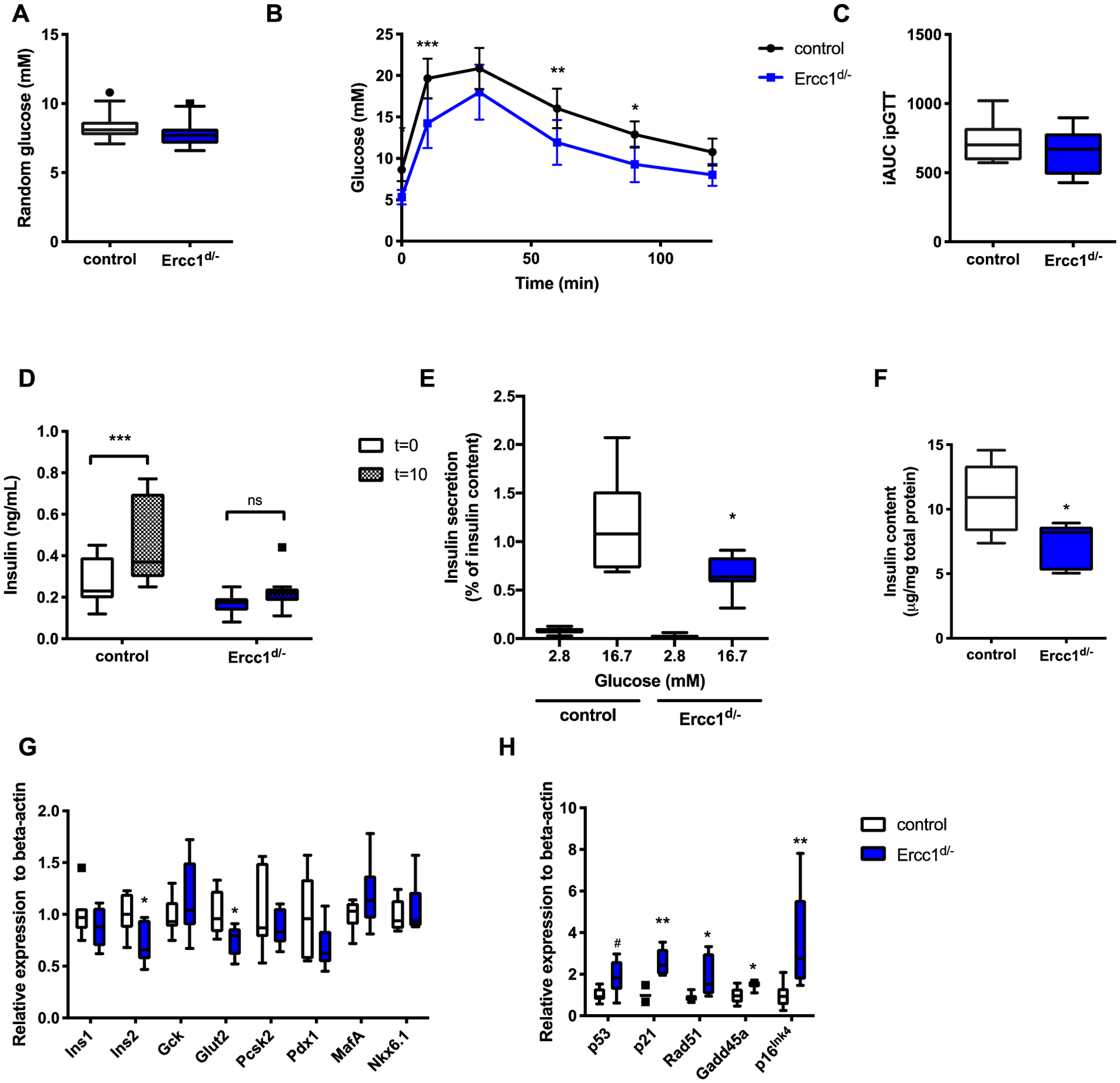
Decreased beta-cell function in *Ercc1*^*d*/−^ mice. (A) Random blood glucose levels were measured in 12 week old *Ercc1*^*d*/−^ and controls (n = 15 mice per group). (B) Oral glucose tolerance test was performed in *Ercc1*^*d*/−^ and control mice (n = 8 mice per group) after 10 h of fasting using 2 g/kg glucose. (C) Incremental area under the curve (iAUC) of the same experiment. (D) Insulin levels of *Ercc1*^*d*/−^ and controls before and after the administrations of the glucose bolus. (E) Glucose-stimulated insulin secretion was measured in isolated islets from of *Ercc1*^*d*/−^ and controls (n = 8 mice per group). (F) Islet insulin content was measured by ELISA in samples isolated from *Ercc1*^*d*/−^ and controls (n = 6–8 mice per group). (G) Relative expression of genes involved in glucose metabolism and (H) DNA damage in isolated islets from of *Ercc1*^*d*/−^ and controls (n = 6–7 mice per group). *P* < 0.05*, *P* < 0.01**, *p* < 0.001***.

Despite the differences in fasting glucose levels, random fed blood glucose levels were normal in *Ercc1*^*d*/−^ mice (Fig. 4A). Glucose tolerance tests in fasted mice revealed an overall reduced glucose levels in *Ercc1*^*d*/−^ mice as compared to controls (Fig. 4B). However, the incremental AUC was no different between groups (Fig. 4C). Although the increase in blood glucose levels after glucose challenge was similar between *Ercc1*^*d*/−^ and control mice, *Ercc1*^*d*/−^ mice failed to increase the levels of blood insulin 10 min after the administration of the glucose bolus (Fig. 4D), indicating reduced beta-cell function. To further assess beta-cell function, insulin secretion was measured *ex vivo* using isolated islets from *Ercc1*^*d*/−^ and control mice. Insulin secretion in response to 16.7 mM glucose administration was significantly blunted in islets from *Ercc1*^*d*/−^ mice (Fig. 4E). This is consistent with their decreased insulin content (Fig. 4F) and reduced expression of *Ins2* (Fig. 4G) in islets from these mice. In addition, the expression of *Glut2* (*SLC2A2*), the main glucose transporter in murine beta-cells, was also decreased in *Ercc1*^*d*/−^ islets. Other genes involved in glucose metabolism, insulin processing, or beta-cell identity were unaffected (Fig. 4G). Consistent with previous reports of increased DNA damage accumulation in several tissues of *Ercc1*^*d*/−^ mice [13], *Ercc1*^*d*/−^ islets showed increased expression of the DNA damage and senescence markers *p53*, *p21 (Cdkn1a)*, *Rad51*, *Gadd45a* and *p16 (Cdkn2a)* (Fig. 4H).

### 3.4. Ercc1^d/−^ mice display decreased beta-cell area and increased susceptibility to islet cell apoptosis

**Fig. 5.**
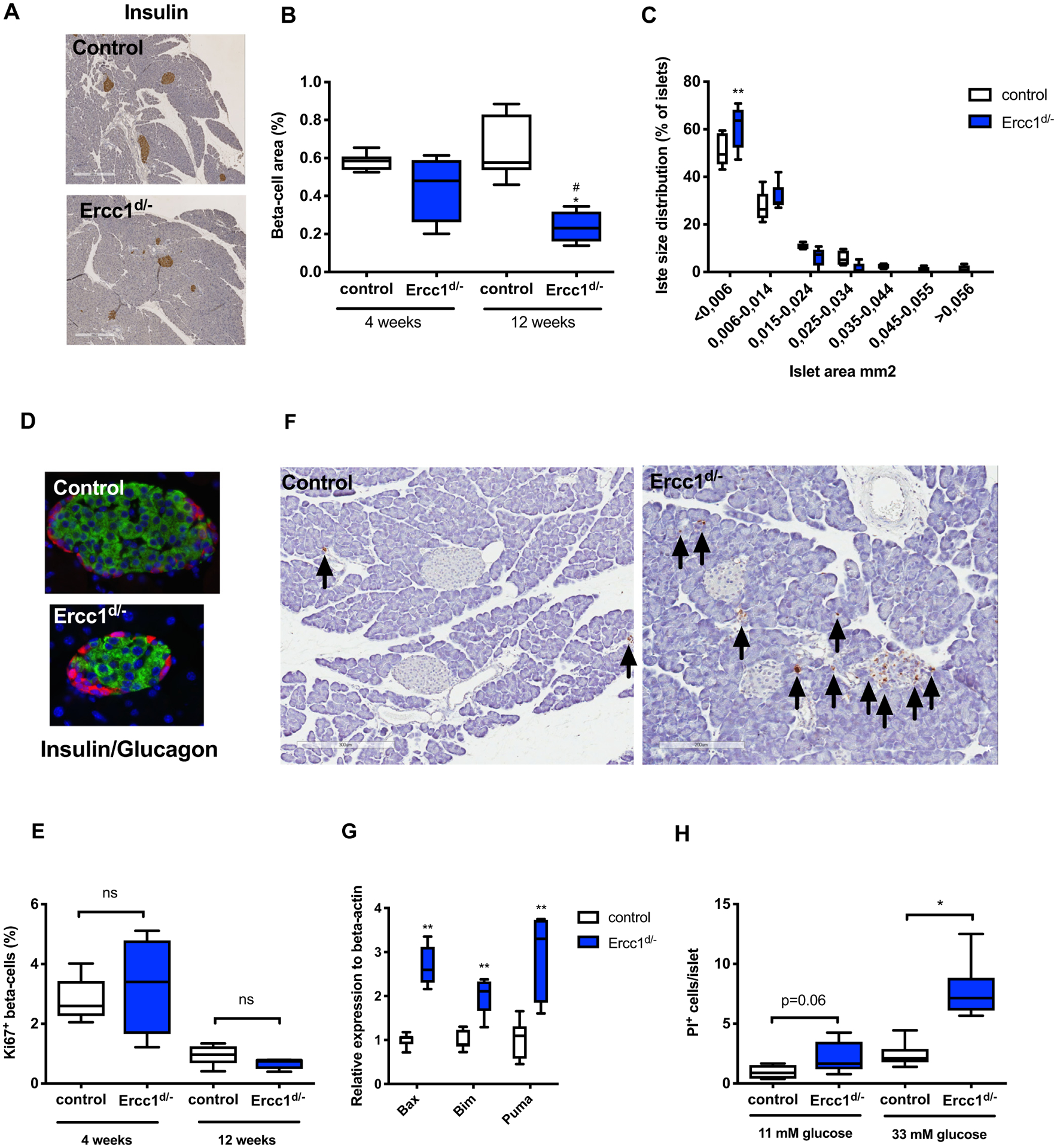
Reduced *Ercc1* expression leads to decreased beta-cell area and increased susceptibility to beta-cell apoptosis. (A) Representative image of insulin staining in the pancreata of *Ercc1*^*d*/−^ mice and age-matched controls (B) Beta-cell area of *Ercc1*^*d*/−^ mice and littermate controls was quantified by immunohistochemistry (n = 5–9 mice per group). (C) Islet size distribution in 12-week-old *Ercc1*^*d*/−^ mice and controls (n = 5–8 mice per group). (D) Representative images of islet morphology after immunofluorescent staining of insulin (green) and glucagon (red). (E) Beta-cell proliferation was determined by the quantification of the Ki67^+^, a marker of S-phase, in sections from pancreata from *Ercc1*^*d*/−^ mice and age-matched controls (n = 5 mice per group). (F) TUNEL staining to detect apoptotic cells in the pancreata of 12 week old *Ercc1*^*d*/−^ mice and controls. (G) Relative expression of genes involved in p53 induced apoptosis in isolated islets from of *Ercc1*^*d*/−^ and controls (n = 6–7 mice per group). (H) Susceptibility to glucose-induced apoptosis was assessed *ex vivo* in islets isolated from *Ercc1*^*d*/−^ mice and controls (n = 6–9 mice per group) exposed to 11 or 33 mM of glucose. *P* < 0.05*, *P* < 0.01** compared to control mice and p < 0.05^#^ compared to 4-week-old *Ercc1*^*d*/−^ mice.

Increased insulin sensitivity in mice is associated with reduced beta-cell mass [33]. We therefore quantified the beta-cell area of *Ercc1*^*d*/−^ mice using immunohistochemistry. Beta-cell area was significantly reduced in 12-week-old *Ercc1*^*d*/−^ mice compared to controls (Fig. 5A,B). This was not attributable to decreased body weight, as beta-cell area was also reduced compared to weight-matched control mice (0.66 ± 0.11% in control *vs*. 0.25 ± 0.09% beta-cell area in *Ercc1*^*d*/−^ mice, *P* < 0.001). Beta-cell area was similar between 4-week-old *Ercc1*^*d*/−^ and control mice (Fig. 5B), indicating normal embryonic development of endocrine pancreas in *Ercc1*^*d*/−^ mice. By 12-weeks of age, mutant mice had a significantly increased proportion of smaller islets compared to control mice (Fig. 5C). Islets of *Ercc1*^*d*/−^ mice had normal architecture (beta-cell and alpha-cell distribution) with a solid core of insulin-producing beta-cells surrounded by glucagon producing cells (Fig. 5D).

Reduced beta-cell area could be caused by decreased beta-cell proliferation or survival. Staining for the proliferation marker Ki67 and insulin revealed increased beta-cell proliferation in 4-week-old mice compared to 12-week-old mice, but did not reveal any differences between genotypes (Fig. 5E). TUNEL staining revealed increased apoptosis in the pancreas of *Ercc1*^*d*/−^ mice relative to age-matched controls, including the endocrine islets (Fig. 5F). Isolated islets of *Ercc1*^*d*/−^ mice showed increased expression of p53-induced pro-apoptosis genes *Bax*, *Bim* and *Puma* (Fig. 5G). In addition, *ex vivo*, islets of *Ercc1*^*d*/−^ mice were more susceptible to glucotoxic-induced apoptosis (Fig. 5H).

## 4. Discussion

Beta-cells of T2D patients show increased expression of markers of genotoxic stress [34,35] and loss of DNA damage repair or activation of the DDR result in disturbed glucose homeostasis [7–9], suggesting a possible involvement of DNA damage accumulation as a cause of beta-cell dysfunction. Mice deficient in *Ercc1* have been extensively studied due to the accelerated aging phenotype modeling a human progeroid syndrome and have been shown to be a powerful system for identifying health-sustaining interventions [31,36]. Loss of *Ercc1* results in measurable DNA damage accumulation in multiple tissues [13], which is comparable but accelerated compared to wild-type organisms. In this study, we investigated the effects of DNA damage accumulation on glucose metabolism in this model system. Inability to repair DNA damage led to increased expression of DNA damage markers, decreased insulin secretion and increased susceptibility to apoptosis in islets from *Ercc1*^*d*/−^ mice. At the whole organism level, however, *Ercc1*^*d*/−^ mice display increased insulin sensitivity and suppression of the somatotropic axis. We conclude that endogenous DNA damage can be a driver of impaired beta-cell function, yet the response to chronic genotoxic stress improves insulin sensitivity by suppressing the somatotropic axis.

Attenuation of the GH/IGF-1 axis, inducing a shift from growth to somatic maintenance, is associated with longevity and healthy aging [37]. Accelerated aging models due to defects in TC-NER display suppression of the somatotropic axis [14,15,38]. Mechanistically, it has been shown that persistent DNA damage in transcriptionally-active regions leads to stalling of RNA polymerase II, which in turn provides the activation of the DDR and attenuation of the GH/IGF1 axis [17]. Reduced somatotropic signaling is associated with extended lifespan and reduced tumor growth [37], so somatotropic attenuation in the presence of persistent DNA damage enables survival under adverse conditions. Our study provides evidence that endogenous genotoxic stress does trigger suppression of the somatotropic axis and in turn, suppression of this axis is protective against genotoxic stress. The latter has implications for cancer patients treated with high dose genotoxins or ionizing radiation.

The insulin pathway is intricately linked to the somatotropic axis as insulin and IGF-1 receptors exhibit marked structural and functional homology, reflecting common evolutionary origin [39]. Mouse models and humans with mutations suppressing the somatotropic axis show decreased body size and increased insulin sensitivity [40], which ultimately protects against diet-induced obesity, insulin resistance, and glucose intolerance. This increased insulin sensitivity could explain the low incidence of diabetes in patients with defective TC-NER, despite having a progressive aging phenotype. A review of 140 Cockayne syndrome patients revealed no patients with diabetes [16] and in the literature only 6 CS patients with T2D have been reported [41,42].

Previous studies showed that DNA damage accumulation in multiple tissues of *Ercc1*^*d*/−^ mice is associated with liver and adipocyte dysfunction [43,44]. Our data reveals that reduced *Ercc1* expression leads to reduced beta-cell area and function. One explanation for the reduced beta-cell area could be the decreased demand for insulin, as beta-cell mass is influenced by insulin sensitivity [33,45]. However, we also showed increased DNA damage markers, such as *p53*, *p21*, *p16* and *Rad51*, in islets of *Ercc1*^*d*/−^ mice, indicating that beta-cells with reduced *Ercc1* expression accumulate DNA damage. This stochastic DNA damage accumulation is associated with increased expression of p53-induced apoptotic genes. Islets of *Ercc1*^*d*/−^ mice show increased cell death both *in vivo* and *ex vivo* under glucotoxic conditions, indicating loss of *Ercc1* has a negative impact on beta-cell survival. It has been reported that other DNA repair deficiencies such as loss of double-strand break (DSB) repair, result in severe early-onset diabetic phenotype. This phenotype is associated with decreased beta-cell proliferation, and occurs even when these mice have normal insulin sensitivity [11]. Genetic instability due to loss of the pituitary tumor transforming gene (Pttg), which encodes a securing protein critical in regulating chromosome separation, also results in decreased beta-cell mass due to increased beta-cell apoptosis and decreased proliferation [46]. Young, insulin-sensitive *Pttg*^−**/−^ mice do not show changes in glucose metabolism. However, older male *Pttg*^−**/−^ mice with insulin resistance become diabetic [47]. Collectively, these data suggest that improved insulin sensitivity in *Ercc1*^*d*/−^ mice masks the detrimental impact of DNA damage accumulation on beta-cells and potentially protects against a diabetic phenotype. This emphasizes the importance of insulin sensitivity in glucose control and suggests that targeting insulin sensitivity could be a feasible strategy to prevent metabolic dysregulation even in light of beta-cell dysfunction.

Our study provides a detailed characterization of changes in insulin sensitivity in *Ercc1*^*d*/−^ mice and the mechanisms that drives these changes. Importantly, this is the first study that examines the role of *Ercc1* in regulation of pancreatic islet senescence, function and mass. However, there are still many unanswered questions. Increased glucose disposal could be due to changes in energy demand or metabolic flexibility. Additional experiments looking into nutrient fluxes, tissue-specific insulin signaling and mitochondrial function, and hormonal control during fasting and feeding are needed to further explore the mechanisms behind the increased insulin sensitivity in *Ercc1*^*d*/−^ mice. Furthermore, the detrimental effect of DNA damage accumulation on beta-cell function and its role in the pathogenesis of type 2 diabetes remains to be further elucidated. We realize that the impact of *Ercc1* deficiency on the GH/IGF-1 axis, influencing growth, body size and composition, complicate these studies. For this reason, future studies should focus on using inducible, tissue-specific DNA repair knockout models, with normal or decreased insulin sensitivity, for the analysis of the impact of DNA damage accumulation on insulin sensitivity, beta-cell mass, function and the role in the pathogenesis of type 2 diabetes.

## Abbreviations:

DDR: DNA damage response
*Fbp1*: *fructose-1,6-biphosphatase 1*
GH: growth hormone
*G6pc*: *glucose-6-phosphatase*
*G6pt*: *glucose-6-phosphate transporter*
IGF1: insulin like growth factor
*Pc*: *pyruvate carboxylase*
PTTG: pituitary tumor transforming gene
*Pygl*: *glycogen phosphorylase*
RER: respiratory exchange ratio
ROS: reactive oxygen species
TC-NER: transcription-coupled nucleotide excision repair
